# Dataset on sociability, cognitive function, gene and protein expression of molecules involved in social behavior, reward system and synapse function following *early-life status epilepticus in* Wistar rats

**DOI:** 10.1016/j.dib.2020.105819

**Published:** 2020-06-07

**Authors:** Ana Miriã Pacífico, Samuel P. Batista, Fernanda T. Ribeiro, Pedro B. dos Santos, Gabriel Bruno Silveira, Bruna Pascarelli Pedrico do Nascimento, Eduardo Dias Junior, Geraldo Henrique L. Barbosa, Miriam Oliveira Ribeiro, Sergio Gomes da Silva, Roberta M. Cysneiros

**Affiliations:** a Developmental Disabilities Graduate Program. Mackenzie Presbyterian University, São Paulo, Brazil. Rua da Consolação, 930. Prédio 28, CEP 01302-907 São Paulo, SP, Brazil; b Hospital do Câncer de Muriaé - Fundação Cristiano Varella, Muriaé, Brazil; c Centro Universitário UNIFAMINAS, Muriaé, Brazil

**Keywords:** Early-life *status epilepticus*, Pilocarpine, Sociability, Motivation, Cognition, Oxytocin

## Abstract

Early-life *status epilepticus* produces deficit in social interaction and vocalization, enhances anxiety, no cognitive impairment and alters functional connectivity within the hippocampus (CA3-CA1) and between the hippocampus and prefrontal cortex [1], [2], [3], [4], [5], [6], [7], [8], [9], [10], [11], [12], but the underlying mechanisms remain unknown. This data article contains behavioral and molecular data of the adult male Wistar rats subjected to early life pilocarpine-induced seizures. Animal's behaviors were assessed to social memory and social motivation, working and reference memories and cognitive flexibility. The brain tissues (hypothalamus, hippocampus, amygdala, and striatum) were probed to gene and protein expression of molecules related to social behavior, reward system and synaptic function.

Specifications Table

**Table:** 

Subject	Biology
Specific subject area	Behavioral Neuroscience
Type of data	Figure
How data were acquired	Data were collected using a circular arena, Barnes maze, Octagonal maze (Insight Ltda, Brazil), qPCR using an Applied Biosystem Step one 48 wells, Elisa (Biotek Elx808), GraphPad Prism 8.3.1.
Data format	Raw and Analyzed
Parameters for data collection	Adults animals subjected to early life status epilepticus were submitted to behavioral tests: Social recognition paradigm to evaluate social memory and social motivation. Octagonal and Barnes maze were used to evaluate cognitive function. Brain tissue was processed to gene and protein expression of molecules involved in social behavior, reward system and synapse function.
Description of data collection	The behavioral data were always collected in the same room after 1 h of habituation to the environment. After the behavioral test animals were anesthetized with urethane (1.2 g/kg) and killed by decapitation. The brain structures were dissected and immediately frozen in liquid nitrogen. The tissues were processed according to standard protocols provided by manufacturers.
Data source location	São Paulo, SP Brazil
Data accessibility	Mendeley Data Doi: 10.17632/jk926nfcdr.4 Direct URL to data: http://dx.doi.org/10.17632/jk926nfcdr.4

**Value of the Data**

• The datasets are useful to further research investigating causative association between early life seizures and chronic socialization abnormalities.
• The data provide information that social decision-making network is vulnerable to seizures
• The data provide information that early life pilocarpine-induced seizures reduce the time of social interaction and improve working memory and cognitive flexibility.
• The data provide information that early life pilocarpine-induced seizures increase the oxytocin in hypothalamus and decrease the expression of its receptor in hippocampus.

## 1 Data description

**Fig. 1:**
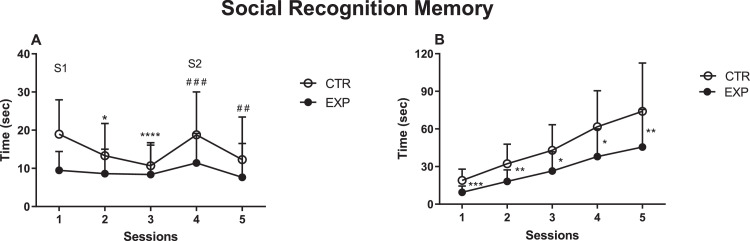
Time spent investigating social stimuli is shown as mean ± SD. In graph A, EXP animals spent lower time investigating S1 compared to the control group and no increase in time was noticed as S1 was replaced by S2. In the cumulative frequency curve (B), exp animals spent lower time investigating social stimuli since the first exposition. A, *, *p* = 0.0116, ****, *p* < 0.0001, ###, *p* = 0.0003; ##, *p* = 0.0043. B, *, *p* = 0.0023; **, *p* = 0.0012; ***, *p* = 0.0007.

In social memory paradigm, the investigation toward the same social stimulus (S1) decreased across the trials 1 to 3 (F (2, 62) = 7.811, *p* = 0.0009, Fig. 1A) only for control group, and it increased from trial 3 to 4 (*t* = 5.874, *p* = 0.0003), as the familiar was replaced by a novel stimulus (S2). In contrast, the experimental group, since the first encounter, spent less time investigating the conspecific, (F (1, 31) = 6.973, *p* = 0.0128). The significant effect of interaction between group versus sessions (F (2, 62) = 4.497, *p* = 0.015), suggests that the experimental group spent lower time in social activity as compared to control. In order to analyze the motivation for social encounter, the time of investigation was expressed as cumulative frequency curve, Fig. 1B. Indeed, experimental group exhibited lower investigation than control group since the first exposition to social stimulus (*t* = 8.01 df=29, *p* = 0.0007), reaching the maximum total time of 45.57 ± 29.94 s against 74.02 ± 38.56 s of control (*t* = 3.32 df=31, *p* = 0.0023).

**Fig. 2:**
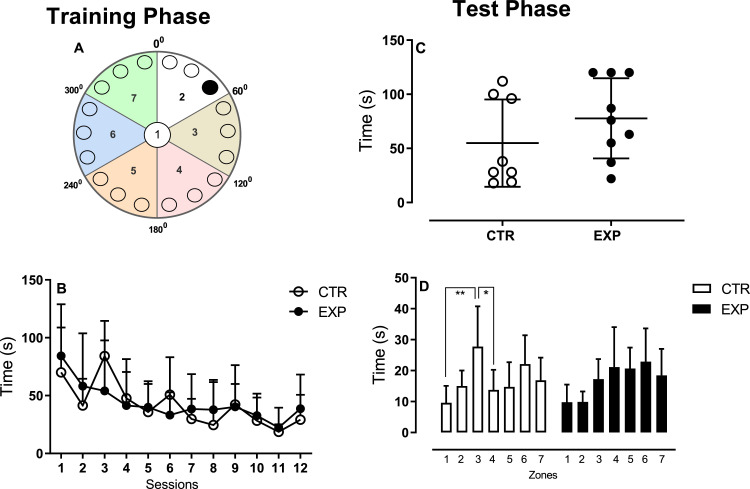
Barnes maze task. Fig A illustrates the maze and its division in zones. The dark circle represents the escape cage location. The data are represented as mean ± SD. Both groups spent a similar time to get into the escape cage (B) or to find the escape hole (C). In D, the control group spent higher time around the target zone and the experimental group distributed its time more evenly among the zones. **, *p* = 0.0012; *, *p* = 0.0424.

In training phase of the Barnes Maze task, the latency to enter into the escape cage decreased across sessions (F (11,165)= 5.25, *p* < 0.0001) for both groups but did not differ between them (F(1165)= 0.11, *p* = 0.73), Fig. 2B. In the test phase, when the escape cage was removed, the time to find the escape hole did not differ between groups (*t* = 1.087, df=7, *p* = 0.31), Fig. 2C. In this phase, as animals were trying to reach an escape route (Fig. 2D), it was noted an effect of interaction between group and zones F (6, 105) = 2.362, *p* = 0.0352), enlightening that experimental group distributed more evenly the time spent among the zones and control animals stayed next to the target hole.

**Fig. 3:**
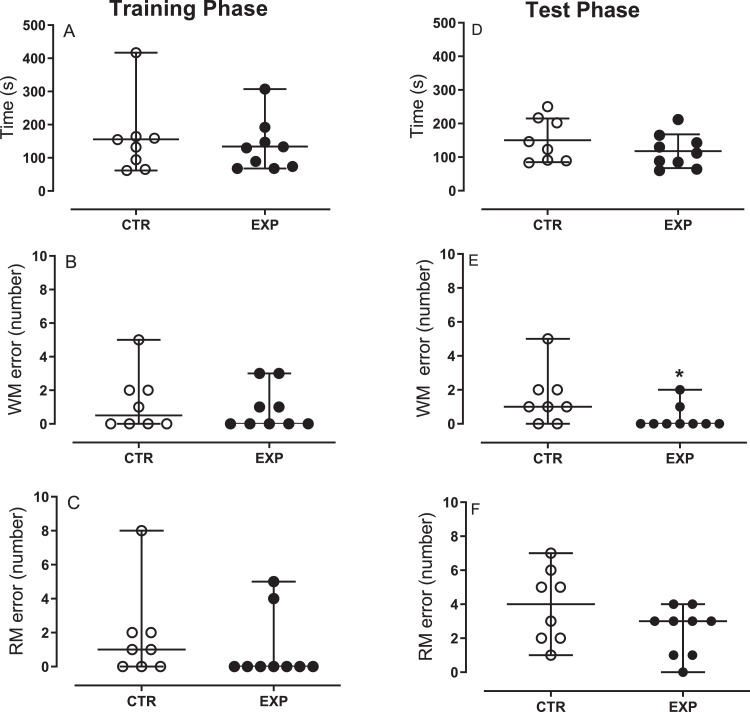
Octagonal maze task. The data are expressed as median ± range, except D, expressed as mean ± SD. During the test phase the experimental group exhibited a lesser number of WM errors than control. *, *p* = *p* = 0.045. WM= working memory, RM= reference memory.

In the octagonal maze task, time spent to complete the task did not differ between groups in training (TRP (*U* = 33, *p* = 0.81) nor in test phases (TP) (*t* = 1.160, *p* = 0.26). Experimental group exhibited less working memory errors during TP (*U* = 16.50, *p* = 0.045), with no difference in reference memory errors in TRP (*U* = 24.50, *p* = 0.2353) nor in TP (*t* = 1.62, *p* = 0.12), Fig. 3.

**Fig. 4:**
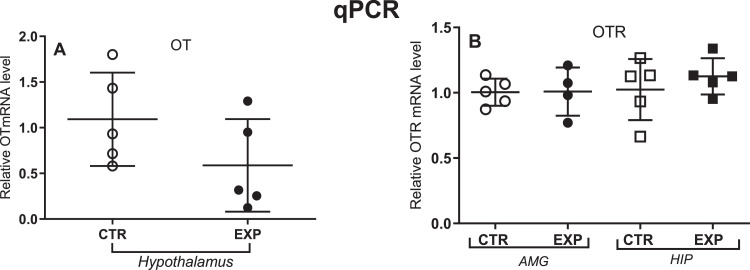
Relative gene expression for Oxytocin (A) and Oxytocin receptor (B) expressed as mean ± SD. Gapdh was used as a constitutive housekeeping gene. The expression of OTR gene decreased in the experimental hippocampus. AMG= amygdala and HIP= hippocampus.

The relative gene expression for Oxytocin (OT) in hypothalamus did not differ between groups, Fig. 4A, as well as for Oxytocin receptor (OTR) in hippocampus nor in amygdala, Fig. 4B.

**Fig. 5:**
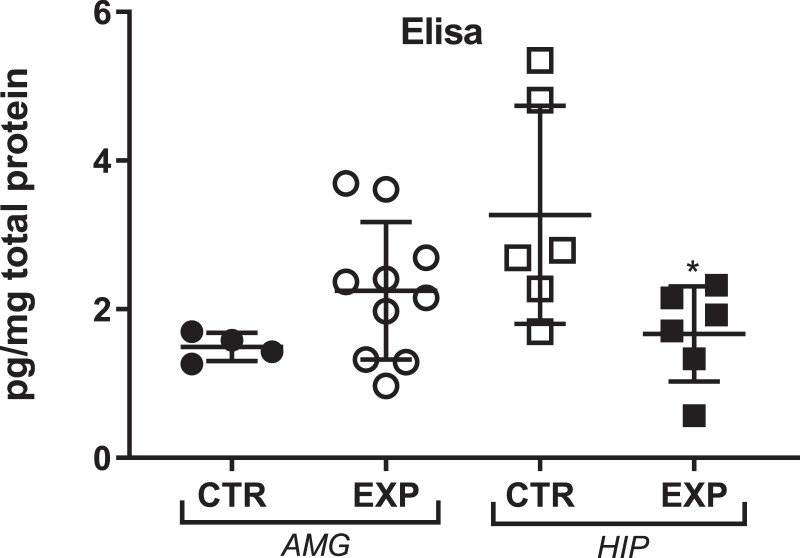
Quantification of Oxytocin receptor (OTR) in amygdala (AMG) and hippocampus (HIP) expressed as mean + SD. The amount of OTR was significantly reduced in hippocampus of experimental group (*, *p* = 0.034). AMG= amygdala; HIP= hippocampus.

In the experimental hippocampus, the protein expression for OTR was significantly reduced as compared to control (*t* = 2.45, df = 10, *p* = 0.034) but no difference was noted in amygdala, Fig. 5.

**Fig. 6:**
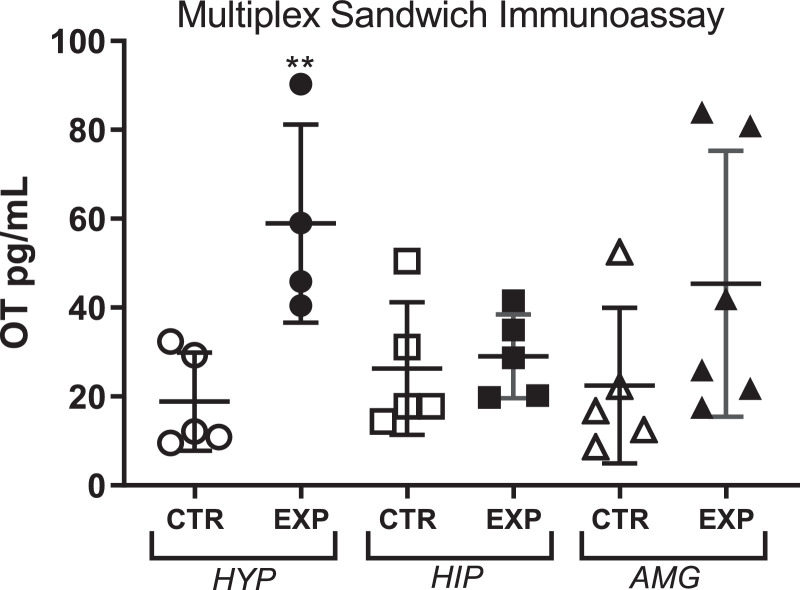
Levels of Oxytocin (OT) in hypothalamus (HYP), hippocampus (HIP) and amygdala (AMG) expressed as mean ± SD. The OT level was significantly higher in experimental hypothalamus. **, *p* = 0.009.

In the experimental group, the oxytocin levels accumulated in hypothalamus were 3.1 times higher as compared to control, with no difference in hippocampus nor in amygdala, Fig. 6. 9

In addition, genes related to the reward system and to synapse function were also probed. In striatum, the relative gene expression for DRD1 and DRD2 receptors did not differ between groups (1.01 ± 0.17 × 1.23 ± 0.9, *t* = 0.4844, df = 6, NS and 1.06 ± 0.43 × 1.28 ± 0.73, *t* = 0.5140, df = 6, NS), respectively, as well in hippocampus for NT3 (1.07 ± 0.49 × 0.55 ± 0.16, *t* = 1.998, df = 6, NS) nor for synapsin (1.03 ± 0.32 × 0.71 ± 0.19, *t* = 1.653, df = 6, NS).

## 2 Experimental design, materials, and methods

All procedures were approved by Mackenzie Presbyterian University Ethical Committee (CEUA, 126/05/2015 and 150/12/2016). Newly born Wistar male rats were maintained under controlled conditions (07:00–19:00 h, light/dark cycle; 22–24 °C) with their mothers. Pups’ ages were determined from the day of birth (P0). Animals from 8 litters per group were randomly assigned to sociability test (CTR: 16 animals and EXP: 17 animals) and for cognitive function tasks (CTR: 8 animals and EXP: 9 animals). The brain tissue from those assigned to sociability were used for molecular procedures related to oxytocin (OT) and oxytocin receptor and those assigned to cognitive task were used for molecular procedures related to reward system and synapse function.

### 2.1 *Status Epilepticus* induction

The experimental group received pilocarpine 3.8% (380 mg/kg, i.p) dissolved in saline on P9, which corresponds to a full-term neonate [13], and the control group received saline 0.9% (0.1 mL/10 g). *Status Epilepticus* (SE) started within 3–4 min after pilocarpine injection being characterized by continuous intense body tremor, scratching, clonic movements of forelimbs and head bobbing. Following cessation of SE (ca 4 h) animals returned to their mothers. At 21 days postnatal, 3–4 male animals of each litter were randomly chosen, group housed (4–5 animals per cage).

### 2.2 Social recognition test

The social recognition test was adapted from Guan e Dluzen, 1994. Initially, the animals were habituated for 3 consecutive days lasting 10 min to the apparatus (circular arena, 60 cm diameter x 50 cm height, Insight Ltda., Brazil) concomitantly with an acrylic box (23.5 cm x 21 cm x 32 cm) which walls had holes of 1 cm of diameter an each 0.5 cm. After that, an unfamiliar conspecific with 30 days of age (social stimulus) was introduced into the acrylic box for 3 min. The procedure was repeated 3 times (trial 1 to trial 3) at intervals of 6 min. In the subsequent sessions (trials 4 and 5), a novel social stimulus of the same age as the previous one was used. It was measured the time of exploration toward a social stimulus. At intervals, the animal test was removed from the arena and the apparatus was cleaned with 5% ethanol.

### 2.3 Barnes maze test

The apparatus, elevated 100 cm above the floor, was a circular platform (100 cm in diameter) with 18 circular holes (9.0 cm in diameter) evenly spaced around the periphery. The apparatus was divided in 6 zones: 0–60°, 60–120°, 120–180°, 180–240° and 240–360° and the escape cage was located at the 0–60° during the training phase and removed during the test phase, Fig. 2A. Visual cues of different geometric shapes were placed around the maze to assist in spatial orientation. The procedure was divided into four phases: habituation (1day), training (12 days) and test (1day). At the end of each trial, the arena was cleaned with 5% ethanol and rotated clockwise to prevent other cues from the environment, in addition to geometric shapes, serving as spatial orientation. In the habituation phase, the animal was placed in the center of the maze under a dark box for 30 s and subsequently, the box was removed, and the animal could explore the arena for 3 min. If the animal finds the escape cage the timer was interrupted, and the animal remained in the cage for 1 min. If the animal did not find it within 3 min, the animal was gently guided to the cage and remained there for 1 min. In the training phase, the procedure was similar to the previous one, and the animals had up to 2 min to complete the task. In the test phase, performed 24 h after training session, the escape cage was removed, and it was measured the time to locate the escape hole and the time spent in all zones of the apparatus.

## 3 Octagonal maze test

Testing was carried out in an eight-arm radial maze (Insight LTDA, Brazil) and started 2 days after the habituation period. The habituation occurred for 2 consecutive days. In the first day, animals food deprived, in pairs, explored the maze for 10 min or until the food pellets spread out in the apparatus have been collect. Next day, animals, one at a time, under food restriction were subjected of the same procedure. Visual cues of different geometric shapes were placed around the maze to assist in spatial orientation. The protocol allowed the dissociation between working and reference memories and was divided in training and test phases. In the training phase, all arms were opened, but only four arms were baited with a pellet of cereal covered with dark chocolate. The training phase lasts 10 min or until the animals have collected the food. The test phase was carried out 30 min after training. The following parameters were analyzed: number of entries into baited arms (hits), number of entries into no baited arms (error), number of re-entries into baited arms (error), total number of errors and the time to finalize the task. Entries into no baited arms were considered reference memory errors and re-entered into baited arms was considered working memory errors.

## 4 Brain-Tissue collection

Rats anesthetized with urethane (1.2 g/kg) were decapitated, the brain was quickly dissected and the brain structures (hippocampus, striatum, amygdala and hypothalamus) were immediately frozen in liquid nitrogen and stored at −80 °C until analysis.

## 5 Oxytocin (OT) quantification

OT concentrations were measured in hypothalamus, hippocampus and amygdala. The brain structures were homogenized in RIPA buffer (Millipore, 1:3, w/v), centrifuged (10,000 × *g* at 4 °C for 10 min) and the supernatant was transferred to a polypropylene tube. The quantification was carried out in the supernatant using the Milliplex Rat Magnetic Bead assays (Millipore) with the MagPix instrument (Luminex, Millipore) according to the manufacturer's protocol.

## 6 Oxytocin receptor (OTR) quantification

OTR quantification in hippocampus and amygdala was assayed using a competitive enzyme immunoassay kit (ELISA) from Elabscience (E-EL-R1446). The procedures were performed following the protocol provided by the kit. Briefly, the brain tissue, free of blood, was sonicated with an ultrasonic cell disrupter till, homogenized in lysis buffer (w:*v* = 1: 5), centrifuged for 5 min at 10,000 x g and the supernatant assayed immediately. Samples, standards and blank (100 μL) were loaded in duplicate into each well. The plate was sealed and incubated for 1 h at 37 °C. After incubation, the liquid was removed and then 100 μl of Biotinylated OTR´s Detector Antibody was added. The plate was incubated for 1 h at 37 °C. After incubation, the wells were washed three times, the Avidin-HRP Conjugate (100 μl) was added into each well and the plate was kept for 30 min at 37 °C. After that, the wells were washed 5 times, the TMB substrate (90 μl) was added and the plate was kept at 37 °C for 20 min. The reaction was stopped by addition of 50 μl of stop solution. Immediately, the plate was reading on the spectrometer at 450 nm. The concentration of OTR was determined from the standard curve, multiplied by the dilution factor and normalized by total protein concentration.

## 7 RNA extraction/CDNA synthesis

Frozen tissues were directly homogenized in Trizol reagent (Life Technologies) at a ratio of 1 ml Trizol/100 mg tissue. Briefly, total tissue RNA was extracted using the RNeasy^Ⓡ^ Lipid tissue Mini Kit (Qiagen), following the manufacturer's instructions. RNA was quantified with a NanoDrop 2000 spectrophotometer (Thermo) d 0.5–1.0 μg total RNA was used to produce cDNA using the Transcriptor First Strand cDNA Synthesis Kit (Roche).

## 8 Real-Time PCR

Gene expression analysis of OT, OTR and the house-keeping gene (Gapdh) was performed using TaqMan Gene Expression Assays (assay ID: OT, Rn00564446_g1; OTr, Rn00563503_m1; Gapdh, Rn01775763_g1) following standard procedure: 2 min at 50 °C, 10 min at 95 °C, 40 cycles of 15 s at 95 °C and 1 min at 60 °C. All reactions were performed in triplicate and the average was used.

The mRNA expression of D1R, D2R, NT3, Synapsin I and house-keeping gene (B-actin) were measured (Step-one Applied Biosystem) using PerfeCTa SYBR Green Fastmix Rox (Quantas). The melting curve protocol was performed to verify the specificity of the amplicon generation. Standard curves consisted of 4–5 points of serially diluted mixed experimental and control group cDNA. The primers used for of D1R (Forward:5´-CGGGCTGCAGCGGAGAG-3´, Reverse:5´- TGCCCAGGAGAGTGGACAGG-3´), D2R (Forward: 5´-AGACGATGAGCCGCAGAAAGNT3-3, Reverse: 5´-GCAGCCAGCAGATGATGAAC-3´), Synapsin I (Forward: 5´-TTGTGGGTAGACACGTGCTC-3´, Reverse:5´-TTCCACGATGAGCTGCTTGT-3´, NT3 (Forward:5´-GGGGATTCCACGGACCAAAT-3´, Reverse: 5´-GAGTCGAAGTAGTAGGGCGC-3´) and B-actin (Forward:5´-TTGCTGACAGGATGCAGA-3´, Reverse:5´-ACCAATCCACACAGAGTACTT-3´) were obtained from Exxtend Biotecnologia, Brazil. PCR amplifications followed standard conditions: 5 min at 95 °C, 40 cycles of 10 s at 95 °C and 30 s at 60 °C. The coefficient of correlation was greater than 0.99 for all curves, and the amplification efficiency ranged between 80% and 100%. All reactions were performed in duplicate and the average of the duplicate was used. Relative gene expression was calculated using the 2-ΔΔCT method [14].

## 9 Statistical analysis

The sociability test and Barnes Maze tasks was analyzed by Mixed ANOVA using sessions as within-subjects factor and groups (EXP versus CTR) as between-subjects factor. Significant effects were probed with post-hoc testing (Bonferroni). Octagonal Maze´s parameters were analyzed by Mann-Whitney for independent sample. Data from q-PCR and Elisa were compared using *t*-test for independent sample. *p* values of 0.05 or less were considered as significant.

## Declaration of Competing Interest

The authors declare that they have no known competing financial interests or personal relationships which have, or could be perceived to have, influenced the work reported in this article.

## Appendix Supplementary materials

Image, application 1

Image, application 2

Image, application 3

## References

[1] Castelhano A.S., Scorza F.A., Teixeira M.C., Arida R.M., Cavalheiro E.A., Cysneiros R.M. (2010). Social play impairment following status epilepticus during early development. J. Neural Transm..

[2] Castelhano A.S., Cassane G dos S, Scorza F.A., Cysneiros R.M. (2013). Altered anxiety-related and abnormal social behaviors in rats exposed to early life seizures. Front. Behav. Neurosci..

[3] Lugo J.N., Swann J.W., Anderson A.E. (2014). Early-life seizures result in deficits in social behavior and learning. Exp. Neurol..

[4] Castelhano A.S., Ramos F.O., Scorza F.A., Cysneiros R.M. (2015). Early life seizures in female rats lead to anxiety-related behavior and abnormal social behavior characterized by reduced motivation to novelty and deficit in social discrimination. J. Neural Transm..

[5] Bernard P.B., Castano A.M., Beitzel C.S., Carlson V.B., Benke T.A. (2015). Behavioral changes following a single episode of early-life seizures support the latent development of an autistic phenotype. Epilepsy Behav..

[6] Bernard P.B., Benke T.A. (2015). Early life seizures: evidence for chronic deficits linked to autism and intellectual disability across species and models. Exp. Neurol..

[7] Leite I.S., Castelhano A.S., Cysneiros R.M. (2016). Effect of diazepam on sociability of rats submitted to neonatal seizures. Data Brief.

[8] Reynolds C.D., Smith G., Jefferson T., Lugo J.N. (2016). The effect of early life status epilepticus on ultrasonic vocalizations in mice. Epilepsia.

[9] Reynolds C.D., Nolan S.O., Huebschman J.L., Hodges S.L., Lugo J.N. (2017). Early-life status epilepticus acutely impacts select quantitative and qualitative features of neonatal vocalization behavior: spectrographic and temporal characterizations in C57BL/6 mice. Epilepsy Behav..

[10] Barbosa G.H.L., Batista S.P., dos Santos P.B., Thomaz C.R.C., Scorza F.A., Cysneiros R.M. (2017). Single neonatal status epilepticus does not impair cognitive function in rats. Epilepsy Behav..

[11] Hodges S.L., Reynolds C.D., Nolan S.O., Huebschman J.L., Okoh J.T., Binder M.S., Lugo J.N. (2019). A single early-life seizure results in long-term behavioral changes in the adult Fmr1 knockout mouse. Epilepsy Res..

[12] Mouchati P.R., Barry J.M., Holmes G.L. (2019). Functional brain connectivity in a rodent seizure model of autistic-like behavior. Epilepsy Behav..

[13] Holopainen I.E. (2008). Seizures in the developing brain: cellular and molecular mechanisms of neuronal damage, neurogenesis and cellular reorganization. Neurochem. Int..

[14] Schmittgen T.D., Livak K.J. (2008). Analyzing real-time PCR data by the comparative C(T) method. Nat. Protoc..

