# Supplementary material for: Dataset on sociability, cognitive function, gene and protein expression of molecules involved in social behavior, reward system and synapse function following early-life status epilepticus in Wistar rats
Source: Data Brief. 2020 Jun 7;31:105819. doi: 10.1016/j.dib.2020.105819 (PMC7306614; doi:10.1016/j.dib.2020.105819)
Supplement: Supplementary file 1 [file mmc1.pdf]

UNIVERSIDADE PRESBITERIANA MACKENZIE

DECANATO DE PESQUISA E PÓS-GRADUAÇÃO

COORDENADORIA DE PESQUISA

COMISSÃO DE ÉTICA NO USO DE ANIMAIS

São Paulo, 25 de junho de 2015

À Senhora

Profª. Drª. Roberta Monterazzo Cysneiros

Centro de Ciências Biológicas e da Saúde

Após a análise do projeto de pesquisa **Avaliar o efeito do estado de ansiedade sobre o prejuízo da interação social em ratos expostos ao status epileticus neonatal**, Processo CEUA/UPM Nº 126/05/2015, sob sua responsabilidade e de sua Orientanda ***Ana Miriã Pacífico***, a Comissão de Ética no Uso de Animais da Universidade Presbiteriana Mackenzie informa que os procedimentos encontram-se de acordo com os preceitos da Lei nº 11.794/2008, do Decreto nº 6.899/2009 e das normas do Conselho Nacional de Controle da Experimentação Animal (CONCEA), e **aprovou** os procedimentos éticos do referido projeto.

Dados do projeto de pesquisa:

Vigência - 12/1/2014 a 12/1/2015
Espécie/Linhagem - Rattus norvegicus
Nº de animais - 40
Peso - não especificado
Idade - a partir de 09 dias
Sexo - Machos
Origem dos animais - Biotério da UPM

Solicitamos, por gentileza, que após a conclusão do citado projeto seja encaminhada uma cópia digital, do Relatório Final, para finalizarmos o seu processo nesta Comissão.

Atenciosamente,

Prof. Dr. Elizeu Coutinho de Macedo

Presidente da Comissão de Ética no Uso de Animais

Projeto analisado na Reunião Mensal de junho.
