# Supplementary material for: Dataset on sociability, cognitive function, gene and protein expression of molecules involved in social behavior, reward system and synapse function following early-life status epilepticus in Wistar rats
Source: Data Brief. 2020 Jun 7;31:105819. doi: 10.1016/j.dib.2020.105819 (PMC7306614; doi:10.1016/j.dib.2020.105819)
Supplement: Supplementary file 2 [file mmc2.pdf]

UNIVERSIDADE PRESBITERIANA MACKENZIE

PRÓ-REITORIA DE PESQUISA E PÓS-GRADUAÇÃO

COORDENADORIA DE PESQUISA

COMISSÃO DE ÉTICA NO USO DE ANIMAIS

São Paulo, 22 de dezembro de 2017

À Senhora

Profª. Drª. Roberta Monterazzo Cysneiros

Programa de Pós-Graduação em Distúrbios do Desenvolvimento

Após a análise do adendo ao projeto de pesquisa **Buscar evidências de validade do modelo do Status epilepticus neonatal para estudo dos déficits do neurodesenvolvimento**, Processo CEUA/UPM Nº 150/12/2016, sob sua responsabilidade, a Comissão de Ética no Uso de Animais da Universidade Presbiteriana Mackenzie informa que os procedimentos encontram-se de acordo com os preceitos da Lei nº 11.794/2008, do Decreto nº 6.899/2009 e das normas do Conselho Nacional de Controle da Experimentação Animal (CONCEA), e **aprovou** os procedimentos éticos do referido projeto.

Dados do projeto de pesquisa:

Vigência - 01/2017 a 03/2018
Espécie/Linhagem - *Rattus norvegicus*
Nº de animais - 20
Peso - 20g
Idade - 09 dias / 60 dias
Sexo - Machos
Origem dos animais - Biotério Central da USP

Solicitamos, por gentileza, que após a conclusão do citado projeto seja encaminhada uma cópia digital, do Relatório Final, para finalizarmos o seu processo nesta Comissão.

Atenciosamente,

Prof. Dr. Elizeu Coutinho de Macedo
Presidente da Comissão de Ética no Uso de Animais

Projeto analisado na Reunião Mensal de dezembro.

Campus São Paulo: *Rua da Consolação, 896 - Ed. João Calvino - térreo ● Consolação ● São Paulo - SP ● CEP 01302-907*
Tel. (11) 2114-8144 ● **www.mackenzie.br** ● prpg.pesq.ceua@mackenzie.br
